# Diabetic state of human coronary artery endothelial cells results in altered effects of bone mesenchymal stem cell‐derived extracellular vesicles

**DOI:** 10.14814/phy2.15866

**Published:** 2023-12-19

**Authors:** Cynthia M. Xu, Catherine Karbasiafshar, Rayane Brinck‐Teixeira, Mark Broadwin, Frank W. Sellke, M. Ruhul Abid

**Affiliations:** ^1^ Cardiovascular Research Center, Rhode Island Hospital Providence Rhode Island USA; ^2^ Division of Cardiothoracic Surgery Alpert Medical School of Brown University and Rhode Island Hospital Providence Rhode Island USA

**Keywords:** diabetes, human bone mesenchymal stem cell‐derived extracellular vesicles, human coronary artery endothelial cells, metabolic syndrome

## Abstract

Human bone mesenchymal stem cell‐derived extracellular vesicles (HBMSC‐EV) have been used successfully in animal models of myocardial ischemia, yet have dampened effects in metabolic syndrome through unknown mechanisms. This study demonstrates the basal differences between non‐diabetic human coronary artery endothelial cells (HCAEC) and diabetic HCAEC (DM‐HCAEC), and how these cells respond to the treatment of HBMSC‐EV. HCAEC and DM‐HCAEC were treated with HBMSC‐EV for 6 h. Proteomics, western blot analysis, and tube formation assays were performed. Key metabolic, growth, and stress/starvation cellular responses were significantly altered in DM‐HCAEC in comparison to that of HCAEC at baseline. Proteomics demonstrated increased phosphorus metabolic process and immune pathways and decreased RNA processing and biosynthetic pathways in DM‐HCAEC. Similar to previous in vivo findings, HCAEC responded to the HBMSC‐EV with regenerative and anti‐inflammatory effects through the upregulation of multiple RNA pathways and downregulation of immune cell activation pathways. In contrast, DM‐HCAEC had a significantly diminished response to HBMSC‐EV, likely due to the baseline abnormalities in DM‐HCAEC. To achieve the full benefits of HBMSC‐EV and for a successful transition of this potential therapeutic agent to clinical studies, the abnormalities found in DM‐HCAEC will need to be further studied.

## 1 INTRODUCTION

Human bone marrow mesenchymal stem cell‐derived extracellular vesicles (HBMSC‐EV) have been widely studied in experimental settings as a potential therapeutic for a wide array of diseases that include the neurologic, pulmonary, cardiovascular, hepatic, hematologic, and musculoskeletal systems (Dabrowska et al., 2019; Gowen et al., 2022; Hingert et al., 2020; Kordelas et al., 2014; Nazarie Ignat et al., 2021; Ophelders et al., 2016; Scrimgeour et al., 2020; Zhang et al., 2021). HBMSC‐EVs are heterogeneous, and made of a cocktail of proteins, lipids, messenger RNA, microRNA, small interfering RNA, DNA, hormones, cytokines and growth factors contained within a bilayer phospholipid membrane (Fitzgerald et al., 2018; Jeppesen et al., 2019; Raposo & Stoorvogel, 2013). They have been touted to have both immunomodulatory and regenerative properties, but the mechanisms of their therapeutic effects are not fully understood (Cheng et al., 2020; Gowen et al., 2022; Roefs et al., 2020).

The therapeutic effect of the extracellular vesicles has been seen in many preclinical studies of cardiovascular disease, including improvements in cardiac function, vascular formation, and oxidative stress (Aboulgheit et al., 2021; Gonzalez‐King et al., 2017; Ma et al., 2017; McLeod, 2022; Potz et al., 2018; Qi et al., 2020; Scrimgeour et al., 2019, 2020; Vrijsen et al., 2016). However, the presence of metabolic syndrome presents a significant obstacle to successful treatment of cardiovascular disease, and on its own increases the risk of coronary artery disease, cardiac ischemic disease, microvascular dysfunction, cardiomyopathy, and heart failure (Tune et al., 2017). Additionally, metabolic syndrome decreased the benefits of HBMSC‐EVs—in a porcine model of metabolic syndrome and chronic myocardial ischemia compared to that with no metabolic syndrome, there was a dysregulation of multiple signalling pathways, including collateral vessel formation mechanisms (Aboulgheit et al., 2021).

Diabetes, a widely prevalent condition in the Unites States, results in both micro‐ and macro‐vascular dysfunction through a variety of mechanisms with associated endothelial dysfunction and oxidative stress, causing coronary artery disease, myocardial ischemic disease, hypertension, peripheral vascular disease, retinopathy, end‐stage renal disease, and neuropathy (Bugger & Abel, 2014; Centers for Disease Control and Prevention, 2022; Du et al., 2001; Knapp et al., 2019; Leon & Maddox, 2015; Luo et al., 2008; Soro‐Paavonen et al., 2010; Wautier et al., 1996; Zhang et al., 2000). Thus, endothelial dysfunction presents as a critical target for addressing cardiovascular disease in this patient population. With previous evidence demonstrating decreased benefits of HBMSC‐EVs in metabolic syndrome, this study aims to determine the effects of HBMSC‐EVs on human coronary artery endothelial cells, both normal (HCAEC) and diabetic (DM‐HCAEC). This study demonstrates basal differences between HCAEC and DM‐HCAEC that could interfere with EV function, as well as identifies dysregulated pathways that ultimately can lead to endothelial cell dysfunction.

## 2 METHODS

### 2.1 HBMSC‐EV production and isolation

Human bone marrow mesenchymal stem cells were obtained from Lonza from a male donor (Walkersville, MD, USA, PT‐2501), and were cultured in 150 cm^2^ flasks with Dulbecco's Modified Eagle Medium (Thermo Fisher #11965092) to passage 7. At 80% confluence, the growth media was removed, the cells were washed with Dulbecco's Phosphate Buffered Saline (PBS) (Gibco, Paisley, UK, 14190‐144), and the media was replaced with 15 mL/flask of serum‐free Roswell Park Memorial Institute medium 1640 (Thermo Fisher #11875085). The cells were incubated for 24 h, and then the serum‐free media was collected. The media was centrifuged at 2000× g for 30 min to remove cell debris, then 100,000× g (WX Ultra Centrifuge with Sorvall AH‐629 rotor) for 70 min to isolate the HBMSC‐EVs, then washed with PBS with an additional 70 min centrifuge cycle at 100,000×g. The HBMSC‐EVs were re‐suspended in PBS with 1% dimethylsulfoxide (DMSO), and stored at −80°C until use.

### 2.2 HBMSC‐EV characterization

The size, number and distribution of HBMSC‐EVs was determined by nanoparticle tracking analysis using the NanoSight NS500 (Malvern Instruments, Malvern, UK). The NanoSight was first verified and optimized with control beads (Malvern Instruments). The HMBSC‐EV sample dilutions were prepared in accordance with manufacturer's instructions, and samples were analyzed in triplicates. The HBMSC‐EVs were evaluated by electron microscopy as well. The HBMSC‐EVs were fixed in 2% paraformaldehyde and were placed onto formwar‐coated electron microscope grids. After 20 min, the HBMSC‐EVs were washed with PBS, fixed with 1% glutaraldehyde and contrasted in 4% uranyl acetate. The HBMSC‐EVs were then imaged with the electron microscope (FEI Morgagni 268). The following HBMSC‐EV markers were evaluated on western blot analysis using the following primary antibodies: CD81 (Cell Signaling #52892S, 1:1000), CD9 (Cell Signaling #13403S, 1:1000), Alix (Cell Signaling #92880S, 1:1000), GAPDH (Cell Signaling #97166S, 1:1000), heat shock protein 70 (HSP70) (Cell Signaling #4872T, 1:1000), and albumin (Cell Signaling #4929S, 1:1000). The following secondary antibodies were used: anti‐mouse IgG, HRP‐linked antibody (Cell Signaling, #7076, 1:5000), and anti‐rabbit IgG, HRP‐linked antibody (Cell Signaling, #7074, 1:5000). In a Bis‐Tris protein gel, 10 μg of HBMSC‐EV protein was loaded and the gel was run in MOPS‐SDS running buffer.

### 2.3 HCAEC culture

Male HCAEC and DM‐HCAEC (Type 2) from single donors were obtained from Lonza (CC‐2585 and CC‐2922) and were cultured in Endothelial Cell Growth Medium Bulletkit (Lonza, CC‐3202) per manufacturer instructions. The glucose concentration of culture conditions was 5.5 mM or 99 mg/dL, which were normoglycemic.

### 2.4 HBMSC‐EV treatment of HCAEC

At 70%–80% confluence and passage 6, the regular growth media was removed and replaced with serum‐free starvation media, consisting of 500 mL Endothelial Cell Growth Basal Medium (Lonza, CC‐3121) and 0.5 mL Gentamicin sulfate‐Amphotericin (Lonza, CC‐4083). The cells were incubated with the starvation media for 12 h for cell cycle synchronization. The starvation media was removed and replaced with new starvation media with HBMSC‐EVs suspended at 1×10^8^ particles/mL or starvation media with equal volume vehicle (1% DMSO in PBS). The cells were incubated with the HBMSC‐EVs for 6 h. Afterwards, the HBMSC‐EV suspension was removed, the cells were washed with PBS, and cellular lysates were prepared for proteomics analysis or western blot analysis.

### 2.5 Preparation of sample for proteomics analysis

The cell lysates (four technical replicates per group) were sent to Indiana State University for proteomics analysis for detection of proteins and measurements of protein abundances. To determine the pathways significantly changed, proteins that were significantly different (un‐adjusted *p*‐value <0.055) were identified and entered into the ShinyGo web application (http://bioinformatics.sdstate.edu/go/) for Gene Ontology pathway analysis. This *p*‐value cut‐off for individual proteins was determined by the bioinformatics analysis team at Indiana State University to be the most reasonable to obtain the appropriate number of proteins for pathway analysis.

### 2.6 Western blot analysis

Western blot assay was performed of the cell lysates (five technical replicates per group) by loading 10 μg of cellular protein in a Bis‐Tris protein gel, which was run with MOPS‐SDS running buffer. The following primary antibodies were used: anti‐phospho‐AMP‐activated protein kinase (Thr172, p‐AMPK) (Cell Signaling, #50081, 1:1000), anti‐AMP‐activated protein kinase (AMPK) (Cell Signaling, #5831, 1:1000), anti‐phospho‐p44/42 MAPK (Thr202/Tyr204, p‐ERK) (Cell Signaling, #4370, 1:2000), anti‐p44/42 mitogen‐activated protein kinase (ERK) (Cell Signaling, #4695, 1:1000), anti‐mammalian target of rapamycin (mTOR) (Cell Signaling, #2972, 1:1000), anti‐phospho‐endothelial nitric oxide synthase (Ser1177, p‐eNOS) (Cell Signaling #9571, 1:1000), anti‐endothelial nitric oxide synthase (eNOS) (Cell Signaling #32027, 1:1000), anti‐glutathione peroxidase 1 (GPX1) (Cell Signaling, #3206, 1:1000), anti‐superoxide dismutase 2 (SOD2) (Cell Signaling # 13141, 1:1000), anti‐nuclear factor erythroid 2‐related factor (NRF2) (Cell Signaling, #12721, 1:1000), and GAPDH. The following secondary antibodies were used: anti‐mouse IgG, HRP‐linked antibody (Cell Signaling #7076, 1:5000), and anti‐rabbit IgG, HRP‐linked antibody (Cell Signaling #7074, 1:5000). Band intensities were measured with ImageJ and normalized to GAPDH, and statistical analysis was done using the Shapiro–Wilk test to determine normality and the Mann–Whitney test.

## 3 RESULTS

### 3.1 HBMSC‐EV characterization

**FIGURE 1:**
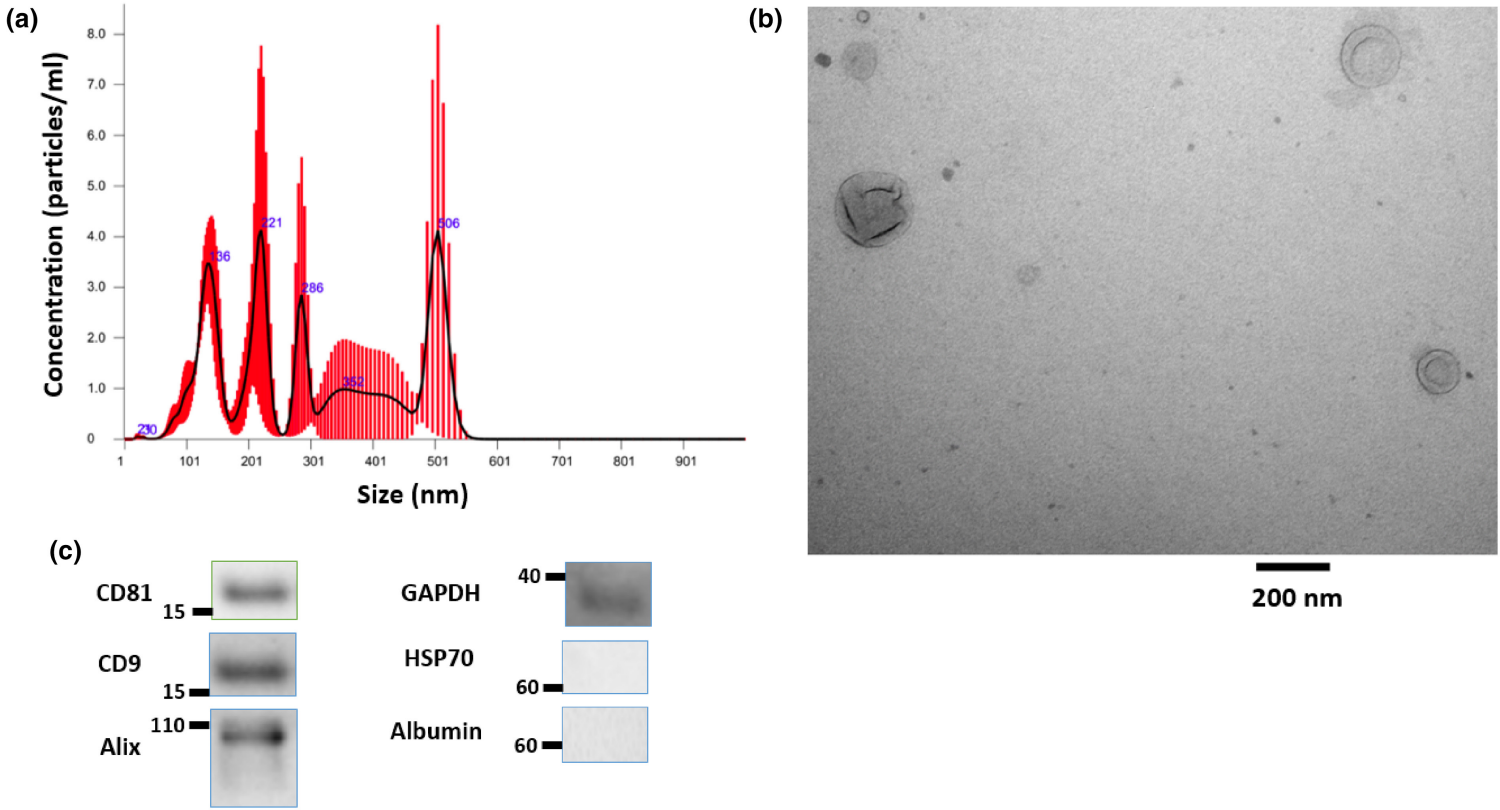
HBMSC‐EV characterizations. (a) NanoSight particle analysis showing size distribution of EVs with corresponding concentrations. (b) Electron microscopy image of EVs (scale bar = 200 nm; magnification 54,800×). (c) Western blot of the HBMSC‐EV demonstrated the presence of transmembrane proteins CD81 and CD9, cytosolic protein Alix and GAPDH. HSP70, a promiscuous cytosolic protein, was not found. Albumin was not identified, which demonstrated purity of the HBMSC‐EV isolation. For western blot characterization of extracellular vesicles, approximately 10 μg of protein was loaded per lane and was interpreted only qualitatively, thus no quantification was done.

The HBMSC‐EV particle sizes and concentrations were quantified using the NanoSight and the average size of the EVs was 228.2 +/− 31.6 nm (Figure 1a). Electron microscopy was used to verify the size and morphology of the HBMSC‐EVs (Figure 1b). Western blot (Figure 1c) demonstrated the presence of typical HBMSC‐EV markers, including transmembrane proteins CD81 and CD9, cytosolic protein Alix and GAPDH. HSP70, a promiscuous cytosolic protein, was not found. The purity of the HBMSC‐EV samples were confirmed by the absence of albumin.

### 3.2 Proteomic analysis demonstrated inherent differences in DM‐HCAEC pathways that may interfere with HBMSC‐EV treatment

**FIGURE 2:**
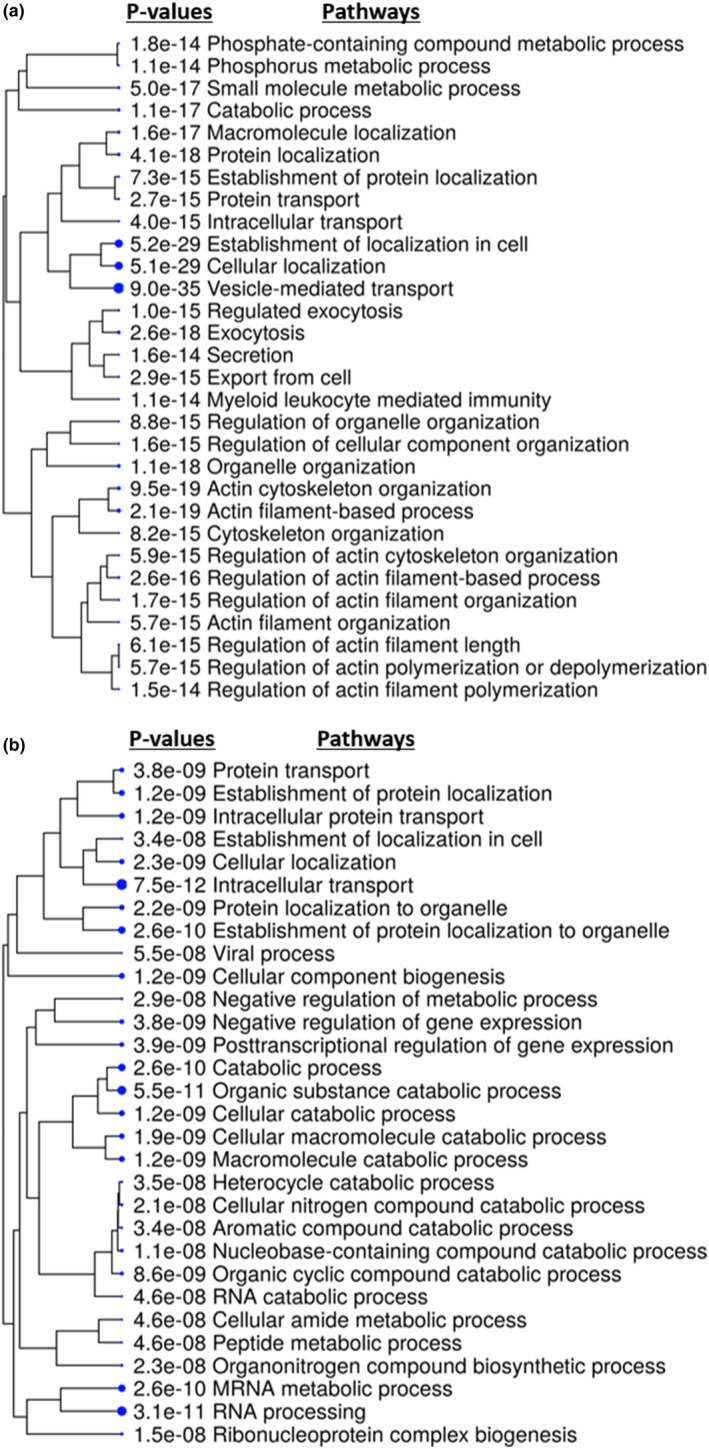
Outputs from ShinyGO showing basal differences in metabolism, inflammation, RNA processing and biogenesis pathways in HCAEC (*n* = 4) and DM‐HCAEC (*n* = 4). (a) Hierarchal clustering tree of pathways up‐regulated in DM‐HCAEC compared to HCAEC. Phosphorus metabolic process and myeloid leukocyte mediated immunity were notably upregulated, with *p*‐values of 1.1 × 10^−14^ and 1.1 × 10^−14^, respectively. The statistical significance of these pathways are noted in the *p*‐values to the left of the respective pathway. Proteins used for the pathway analysis had un‐adjusted *p*‐values < 0.055. (b) Hierarchal clustering tree of pathways down‐regulated in DM‐HCAEC compared to HCAEC. RNA processing and cellular biogenesis pathways were down‐regulated in DM‐HCAEC.

First, the basal differences between HCAEC and DM‐HCAEC were determined (both male donors, five technical replicates per cell line). The expression of over 3000 proteins were significantly changed in DM‐HCAEC. Using Gene Ontology Biological Process pathway analysis, it was found that in DM‐HCAEC, pathways including ones related to phosphate metabolic processes, cellular export/secretion, transport mechanisms, and myeloid leukocyte mediated immunity were found to be up‐regulated (Figure 2a). RNA processing pathways, cellular component biogenesis and ribonucleoprotein complex biogenesis appeared to be down‐regulated (Figure 2b). Source data for this study are openly available at DOI: 10.17632/sc64ncrgp6.2 (Xu et al., 2023).

**FIGURE 3:**
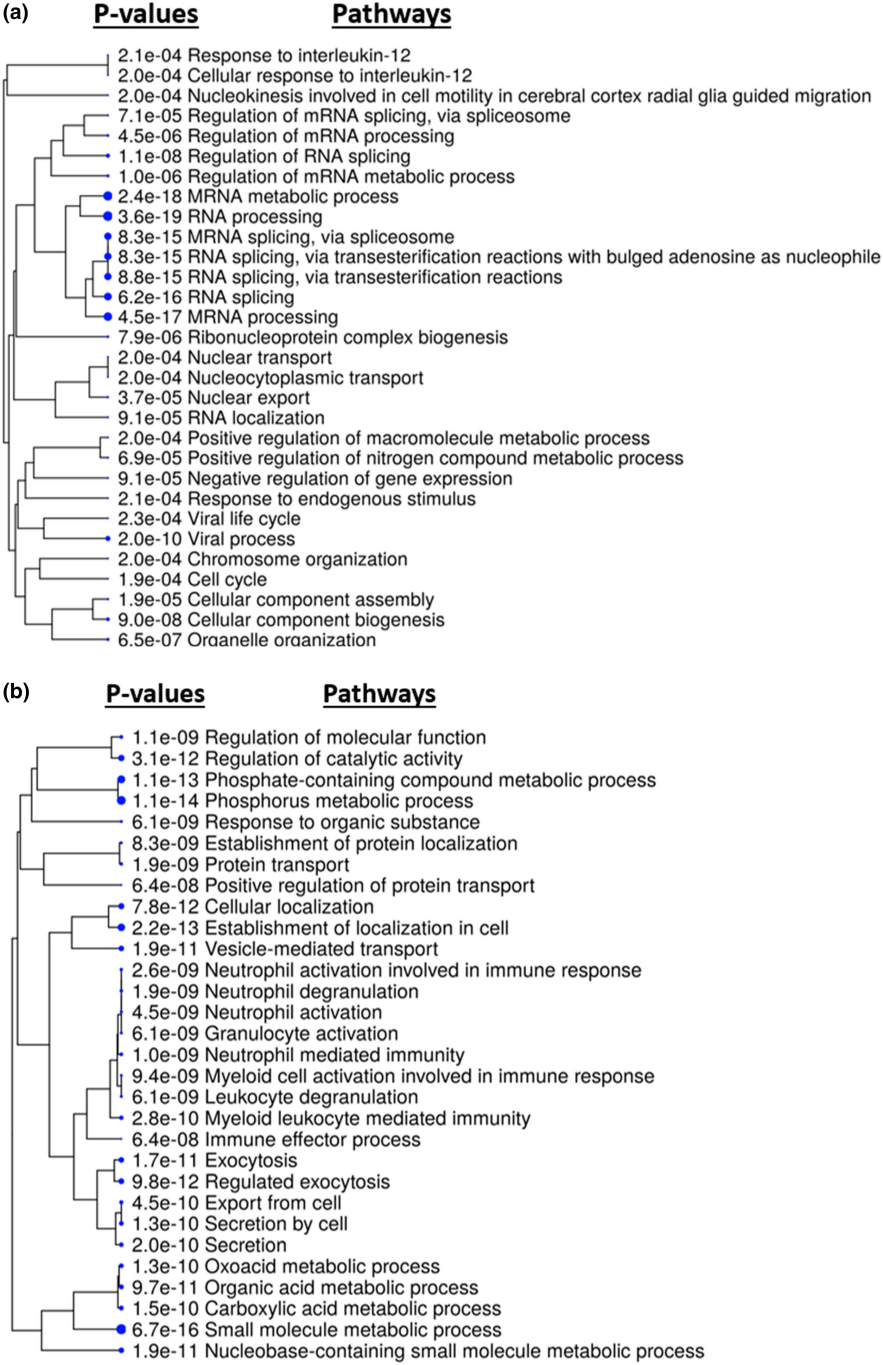
Outputs from ShinyGO showed that HBMSC‐EV treatment of HCAEC (*n* = 4) resulted in increased expression of RNA processing and decreased phosphorus metabolic processes and inflammatory pathways compared to that of vehicle treatment only (*n* = 4). (a) Hierarchal clustering tree of pathways up‐regulated in HCAEC after HMBSC‐EV treatment. The statistical significance of these pathways are denoted on the left of the pathway text. Significant proteins used for pathway analyses had un‐adjusted *p*‐values < 0.055. (b) Hierarchal clustering tree with associated *p*‐values of pathways down‐regulated in HCAEC after HBMSC‐EV treatment.

In HCAEC after HBMSC‐EV treatment, the expression of over 600 proteins were altered. Pathway analysis showed that the pathways that were up‐regulated included ones related to RNA splicing, RNA processing and biogenesis processes (Figure 3a). Pathways that were down‐regulated included related to phosphate metabolic processes, oxoacid/organic acid/carboxylic acid metabolic processes, immune cell activation, and cellular localization (Figure 3b). Source data for this study are openly available at DOI: 10.17632/sc64ncrgp6.2 (Xu et al., 2023).

**FIGURE 4:**
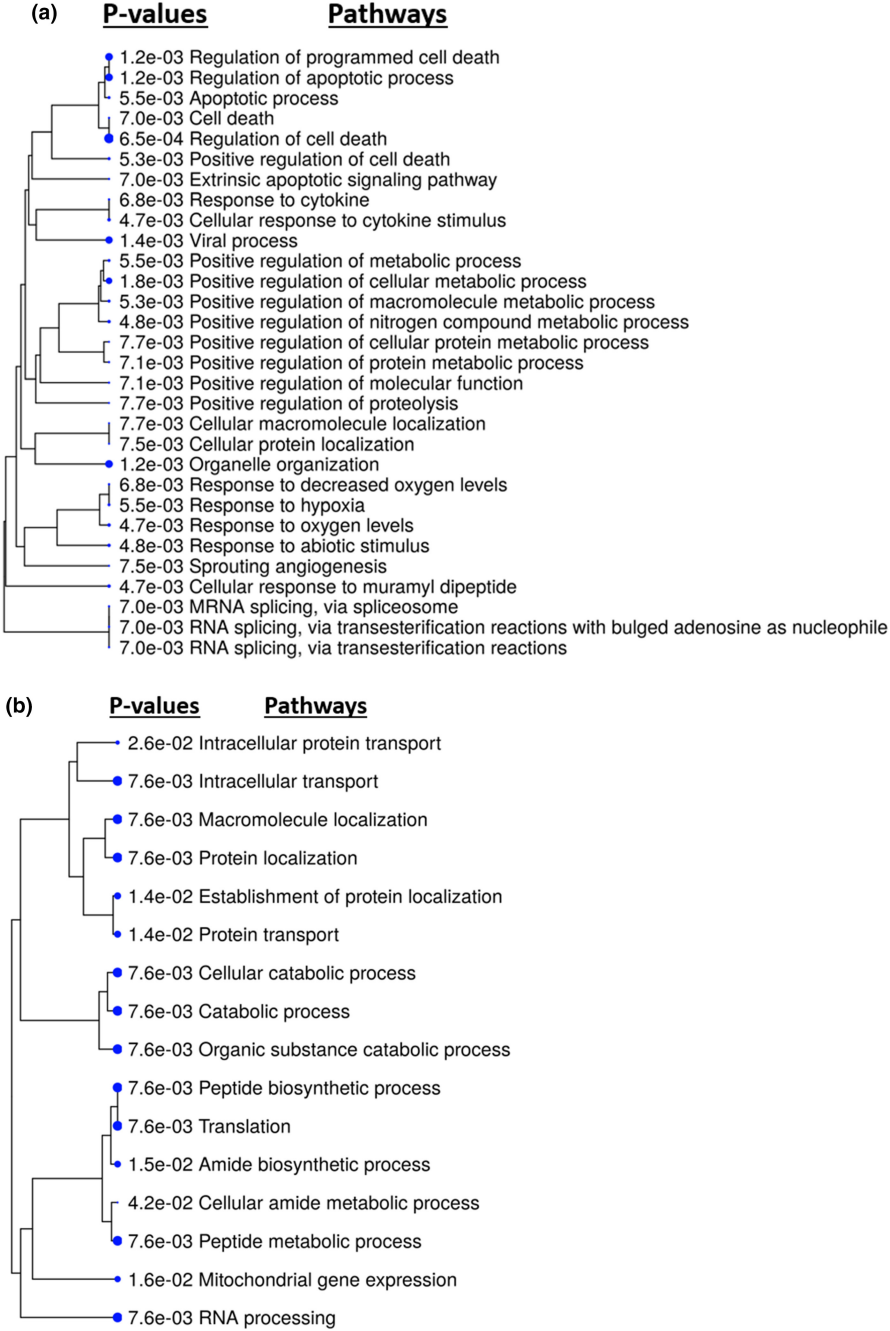
Outputs from ShinyGO showed that HBMSC‐EV treatment of DM‐HCAEC (*n* = 4) resulted in mixed up and down‐regulation of RNA processing pathways and no changes in phosphorus metabolic processes or inflammatory pathways compared to that of vehicle treatment only (*n* = 4). This may be due to baseline differences between HCAEC and DM‐HCAEC. (a) Hierarchal clustering tree of pathways up‐regulated in DM‐HCAEC after HBMSC‐EV treatment. The *p*‐values denoting the statistical significance of these pathways is to the left of the pathway. Regulation of apoptosis and positive regulation of various metabolic processes were up‐regulated, as well as RNA splicing (though to a lesser extent to that of HCAEC). (b) Hierarchal clustering tree with pathways down‐regulated in DM‐HCAEC after HBMSC‐EV treatment. The RNA processing pathway was found to be decreased here, in addition to being up‐regulated, representing a mixed response. No phosphorus metabolic processes or inflammatory pathways were identified here.

In DM‐HCAEC after HBMSC‐EV treatment, the expression of only 264 proteins were changed, and thus fewer pathways were affected. The pathways that were up‐regulated included regulation of apoptosis, RNA splicing (but less than that in HCAEC after EV treatment), sprouting angiogenesis, response to hypoxia, and positive regulation of various metabolic processes (cellular, macromolecule, nitrogen compound, cellular protein, protein) (Figure 4a). The pathways that were down‐regulated after EV treatment included some catabolic processes, mitochondrial gene expression, and protein synthesis (peptide biosynthesis, translation, amide biosynthesis, cellular amide and peptide metabolic process, RNA processing) (Figure 4b). There were no pathway changes in phosphorus metabolic processes and inflammatory pathways. Source data for this study are openly available at DOI: 10.17632/sc64ncrgp6.2 (Xu et al., 2023).

The diminished effects of the HBMSC‐EV in DM‐HCAEC may be due to the basal differences between HCAEC and DM‐HCAEC. While in HCAEC, HBMSC‐EV appeared to increase RNA processing pathways and decrease phosphorus metabolic processes and inflammatory pathways (Figure 3), but all three of these pathways were dysregulated in DM‐HCAEC at baseline. Baseline proteomic analysis showed that in DM‐HCAEC compared to HCAEC there was a decrease in RNA processing and biosynthetic pathways but an up‐regulation of phosphorus metabolic processes and inflammatory pathways (Figure 2). These baseline abnormalities appear to be counteracting to the HBMSC‐EV treatment effects and may have resulted in an overall dampening of HBMSC‐EV effects in DM‐HCAEC.

There appeared to be an overlap of only 27 proteins in the HCAEC and DM‐HCAEC after HBMSC‐EV treatment. Ultimately, the amount of proteins was not enough to complete a pathway analysis.

### 3.3 Western blot showed differences in key metabolic and antioxidant protein expression in HCAEC versus DM‐HCAEC

**FIGURE 5:**
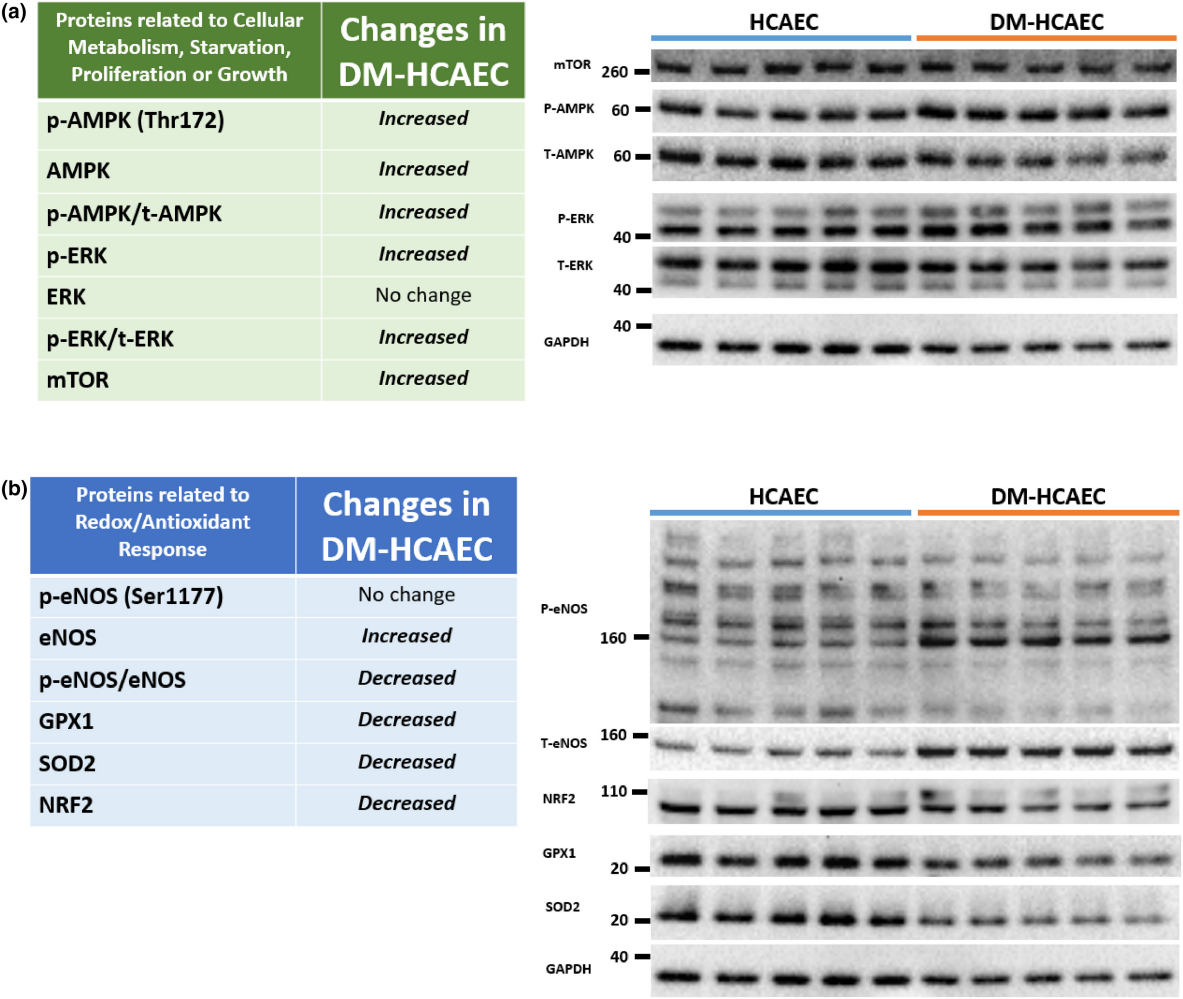
Proteins related to key metabolic pathways and antioxidant regulation were found to be altered in DM‐HCAEC (*n* = 5) compared to HCAEC (*n* = 5) on western blot (right panel), where 10 μg of protein was loaded per well. This data represents findings from one donor per cell type. The Shapiro–Wilk and Mann–Whitney statistical tests were used for analysis. (a) Increase in protein expression of key pathways demonstrate activation of both starvation/stress (AMPK) and proliferative (ERK, mTOR) signalling in DM‐HCAEC. (b) There was an overall decreased expression of multiple antioxidant proteins (GPX1, SOD2, NRF2) in DM‐HCAEC, which may explain the state of increased oxidative stress in DM‐HCAEC.

In conjunction with the proteomic findings, western blot identified several key proteins were significantly different between HCAEC and DM‐HCAEC at baseline. These samples were collected from the same donors and experiment as that of the proteomics experiments. P‐AMPK, AMPK, and p‐AMPK/AMPK, which are activated in the presence of cellular starvation or stress, were significantly increased in DM‐HCAEC compared to that of HCAEC (*p* values were 0.009, 0.05, and 0.009, respectively). This was consistent with the increase of phosphate metabolic processes of the DM‐HCAEC at baseline found on proteomics. Interestingly, proliferative pathways such as p‐ERK, p‐ERK/ERK and mTOR were also significantly increased (*p* values were 0.016, 0.009, and 0.009, respectively) in DM‐HCAEC. The simultaneous and contradictory activation of starvation/stress pathways (AMPK) and proliferative pathways (ERK) may represent the dysfunctional metabolic regulation inherent in DM‐HCAEC. Figure 5a.

Proteins involved in antioxidant/redox pathways were largely decreased in DM‐HCAEC. P‐eNOS was not significantly changed (*p* value = 0.1), but eNOS was significantly increased (*p* value = 0.009) and p‐eNOS/eNOS was significantly decreased (*p* value = 0.009) in DM‐HCAEC. Antioxidants GPX1, SOD2, and NRF2 were all significantly decreased in DM‐HCAEC (*p* values were 0.02, 0.009, and 0.03, respectively). See Figure 5b for result summary. This could explain the state of increased oxidative stress in DM‐HCAEC (Endothelial Dysfunction in Type 2 Diabetes, 2023).

## 4 DISCUSSION

Earlier studies showed dysregulated major signalling pathways in diabetes, but not much is specifically known about the abnormalities in coronary artery endothelial cells (HCAEC) (Blagosklonny, 2019; Coble, 2020; Jeon, 2016; Um et al., 2006; Xu et al., 2016). Our current study demonstrates that a wide range of signalling pathways are altered at baseline in DM‐HACEC, not simply the ones that are related to glucose metabolism. In DM‐HCAEC, profound differences in metabolic, inflammatory, RNA processing, and antioxidant pathways in comparison to HCAEC have been found which may have resulted in the previously observed decreased therapeutic response to HBMSC‐EVs.

DM‐HCAEC had increased activation of AMPK compared to that of HCAEC. This may be due to the impaired ability of glucose uptake of the DM‐HCAEC with resulting increased starvation signalling. Although diabetes has been known to have an inhibited AMPK activity (with resulting decreased glucose uptake and insulin resistance), heightened AMPK activation was identified in DM‐HCAEC in this study (Jeon, 2016). This may be due to endothelial cells' unique preference for glycolysis, rather than oxidative phosphorylation. Conversely, increased (indirect) AMPK activation is a mechanism of many antidiabetic medications to improve glucose uptake—it is unclear what signalling effects these medications have on endothelial cells, though metformin has been shown to improve endothelial function in patients with metabolic syndrome (Coughlan et al., 2014; Jeon, 2016; Vitale et al., 2005; Zhang et al., 2009). Also, the heightened AMPK activation in DM‐HCAEC may have diminished downstream effects—AMPK affects NRF2 indirectly to increase the antioxidant response in the presence of oxidative stress. Interestingly, in DM‐HCAEC, NRF2 expression was decreased as well as the expression of several other antioxidant enzymes, suggesting a plausible mechanism to have increased reactive oxygen species in diabetic endothelial cells where there is found to have decreased antioxidant status (Ashour et al., 1999; Laight et al., 2000; Tüzün et al., 1999).

Despite increased AMPK activation in response to starvation, there was also increased ERK activation in the DM‐HCAEC. This paradoxical signalling involving simultaneous activation of starvation/stress response and proliferative signalling could lead to increased cellular stress. Pathological ERK activation has been implicated in the development of diabetic cardiomyopathy, including contributions to oxidative stress, inflammation, remodeling, and apoptosis (Coble, 2020; Xu et al., 2016). Similarly, we see increased ERK activation in the setting of starvation in DM‐HCAEC. ERK also can downregulate NRF2 in cardiomyocytes, contributing to further oxidative stress and insulin resistance (Tan et al., 2011). MEK/ERK inhibition as a potential therapeutic for diabetic cardiomyopathy has not yet been conclusive (Xu et al., 2016).

mTOR, a major regulator of growth and cellular metabolism, was increased in the DM‐HCAEC, indicating further inappropriate growth signalling and mTOR dysregulation. Increased expression of mTOR in diabetes is known to result in insulin resistance, which has brought about the creation of mTOR inhibitors such as rapamycin for diabetic treatment (Blagosklonny, 2019; Um et al., 2006). Unfortunately, this therapy is not clinically effective—rapamycin improves insulin sensitivity initially but long‐term causes hyperglycemia and insulin resistance (Blagosklonny, 2019).

In HCAEC after HBMSC‐EV treatment, the proteomic findings were consistent with the known properties of HBMSC‐EV—there was up‐regulation of regenerative pathways and the down‐regulation of inflammatory pathways. In DM‐HCAEC however, the expected effects of HBMSC‐EVs were dampened or not present, which may be due to inherent dysregulation of metabolism in DM‐HCAEC. Altogether, HBMSC‐EVs did not achieve their full effect in DM‐HCAEC, which is consistent with previous findings in animal models of metabolic syndrome (Aboulgheit et al., 2021).

Lastly, another aspect to consider is the possibility of variable uptake of HBMSC‐EV in HCAEC and DM‐HCAEC, which could potentially explain the mechanism of decreased HBMSC‐EV response in DM‐HCAEC. EV uptake studies are limited by the minute size of EVs in general, but endothelial cell uptake of EVs has been demonstrated (Durak‐Kozica et al., 2018). Future studies could delve into differences between HCAEC and DM‐HCAEC extracellular vesicle uptake.

In conclusion, HBMSC‐EVs were found to have regenerative and anti‐inflammatory effects on HCAEC, and dampened effects on DM‐HCAEC, which are likely due to the basal abnormalities seen with diabetes. Reversing these metabolic abnormalities may augment the effects of the HBMSC‐EVs in DM‐HCAEC. Additionally, the treatment of diabetes will not lie with glucose control alone, but also the treatment of the metabolic changes present. A limitation in this study was the culture of DM‐HCAEC in normoglycemic conditions which are not the best in vitro simulation of diabetes—there were still however significant differences between the HCAEC and DM‐HCAEC, possibly due to epigenetic changes. Clinically, this could mean that even with adequate blood sugar control, there are irreversible metabolic shifts in diabetic cells that make them more resistant to HBMSC‐EV treatment. Another major limitation is the use of only one donor per cell line, and the donors were only male. This study was exploratory, and it showed to need to explore more the variable effects of HBMSC‐EV. Overcoming these obstacles will be key in successfully transitioning HBMSC‐EVs from preclinical animal models to humans, who have many co‐morbidities in the setting of cardiovascular disease.

## CONFLICT OF INTEREST STATEMENT

The authors have no conflicts of interest or disclosures.

## ETHICS STATEMENT

No human subjects or animals were utilized in this research.

## FUNDING INFORMATION

Funding for this research was provided by the National Heart, Lung, and Blood Institute (NHLBI) 1R01HL133624 (M.R.A.), 2R56HL133624 (M.R.A.) and Rhode Island Foundation grant 1472420231352 (M.R.A); R01HL46716 and R01HL128831‐01A1 (F.W.S.). C.X. was supported by T32 GM065085 (J.A.). M.B. was supported by T32 HL160517 (F.W.S). Funds for proteomics were provided by the IDeA National Resource for Proteomics (R24GM137786) (M.R.A.).

## Supporting information


Data S1.

## References

[phy215866-bib-0001] Aboulgheit, A. , Potz, B. A. , Scrimgeour, L. A. , Karbasiafshar, C. , Shi, G. , Zhang, Z. , Machan, J. T. , Schorl, C. , Brodsky, A. S. , Braga, K. , Pfeiffer, M. , Gao, M. , Cummings, O. , Sodha, N. R. , Abid, M. R. , & Sellke, F. W. (2021). Effects of high fat versus normal diet on extracellular vesicle–induced angiogenesis in a swine model of chronic myocardial ischemia. Journal of the American Heart Association, 10(4), e017437 Available from: https://www.ncbi.nlm.nih.gov/labs/pmc/articles/PMC7955347/ 33559477 10.1161/JAHA.120.017437 PMC7955347

[phy215866-bib-0002] Ashour, M. , Salem, S. , Hassaneen, H. , El‐Gadban, H. , Elwan, N. , Awad, A. , & Basu, T. K. (1999). Antioxidant status and insulin‐dependent diabetes mellitus (IDDM). Journal of Clinical Biochemistry and Nutrition, 26(2), 99–107.

[phy215866-bib-0003] Blagosklonny, M. V. (2019). Fasting and rapamycin: Diabetes versus benevolent glucose intolerance. Cell Death & Disease, 10(8), 1–10. 10.1038/s41419-019-1822-8 PMC6690951 31406105

[phy215866-bib-0004] Bugger, H. , & Abel, E. D. (2014). Molecular mechanisms of diabetic cardiomyopathy. Diabetologia, 57(4), 660–671. 24477973 10.1007/s00125-014-3171-6 PMC3969857

[phy215866-bib-0006] Centers for Disease Control and Prevention . (2022). National Diabetes Statistics Report. Available from: https://www.cdc.gov/diabetes/data/statistics‐report/index.html

[phy215866-bib-0007] Cheng, A. , Choi, D. , Lora, M. , Shum‐Tim, D. , Rak, J. , & Colmegna, I. (2020). Human multipotent mesenchymal stromal cells cytokine priming promotes RAB27B‐regulated secretion of small extracellular vesicles with immunomodulatory cargo. Stem Cell Research & Therapy, 11(1), 539. 33317598 10.1186/s13287-020-02050-6 PMC7734842

[phy215866-bib-0008] Coble, M. (2020). The role of ERK 1/2 proteins in diabetic cardiomyopathy. Available from: https://scholarworks.gvsu.edu/ssd_posters/35

[phy215866-bib-0009] Coughlan, K. A. , Valentine, R. J. , Ruderman, N. B. , & Saha, A. K. (2014). AMPK activation: A therapeutic target for type 2 diabetes? Diabetes, Metabolic Syndrome and Obesity: Targets and Therapy, 24(7), 241–253. 10.2147/DMSO.S43731 PMC4075959 25018645

[phy215866-bib-0010] Dabrowska, S. , Andrzejewska, A. , Strzemecki, D. , Muraca, M. , Janowski, M. , & Lukomska, B. (2019). Human bone marrow mesenchymal stem cell‐derived extracellular vesicles attenuate neuroinflammation evoked by focal brain injury in rats. Journal of Neuroinflammation, 16(1), 216. 31722731 10.1186/s12974-019-1602-5 PMC6852925

[phy215866-bib-0011] Du, X. L. , Edelstein, D. , Dimmeler, S. , Ju, Q. , Sui, C. , & Brownlee, M. (2001). Hyperglycemia inhibits endothelial nitric oxide synthase activity by posttranslational modification at the Akt site. The Journal of Clinical Investigation, 108(9), 1341–1348. 11696579 10.1172/JCI11235 PMC209429

[phy215866-bib-0012] Durak‐Kozica, M. , Baster, Z. , Kubat, K. , & Stępień, E. (2018). 3D visualization of extracellular vesicle uptake by endothelial cells. Cellular & Molecular Biology Letters, 17(23), 57. 10.1186/s11658-018-0123-z PMC6296015 30574165

[phy215866-bib-0013] Endothelial Dysfunction in Type 2 Diabetes . (2023). Targeting Inflammation|IntechOpen [Internet]. Available from: https://www.intechopen.com/chapters/61378

[phy215866-bib-0014] Fitzgerald, W. , Freeman, M. L. , Lederman, M. M. , Vasilieva, E. , Romero, R. , & Margolis, L. (2018). A system of cytokines encapsulated in extracellular vesicles. Scientific Reports, 8(1), 8973. 29895824 10.1038/s41598-018-27190-x PMC5997670

[phy215866-bib-0015] Gonzalez‐King, H. , García, N. A. , Ontoria‐Oviedo, I. , Ciria, M. , Montero, J. A. , & Sepúlveda, P. (2017). Hypoxia inducible factor‐1α potentiates jagged 1‐mediated angiogenesis by mesenchymal stem cell‐derived exosomes. Stem Cells, 35(7), 1747–1759. 28376567 10.1002/stem.2618

[phy215866-bib-0016] Gowen, A. , Shahjin, F. , Chand, S. , Odegaard, K. E. , & Yelamanchili, S. V. (2022). Mesenchymal stem cell‐derived extracellular vesicles: Challenges in clinical applications. Frontiers in Cell and Developmental Biology, 8, 149 Available from: 10.3389/fcell.2020.00149. PMC7080981 32226787

[phy215866-bib-0017] Hingert, D. , Ekström, K. , Aldridge, J. , Crescitelli, R. , & Brisby, H. (2020). Extracellular vesicles from human mesenchymal stem cells expedite chondrogenesis in 3D human degenerative disc cell cultures. Stem Cell Research & Therapy, 11(1), 323. 32727623 10.1186/s13287-020-01832-2 PMC7391655

[phy215866-bib-0018] Jeon, S. M. (2016). Regulation and function of AMPK in physiology and diseases. Experimental & Molecular Medicine, 48(7), e245. 27416781 10.1038/emm.2016.81 PMC4973318

[phy215866-bib-0019] Jeppesen, D. K. , Fenix, A. M. , Franklin, J. L. , Higginbotham, J. N. , Zhang, Q. , Zimmerman, L. J. , Liebler, D. C. , Ping, J. , Liu, Q. , Evans, R. , Fissell, W. H. , Patton, J. G. , Rome, L. H. , Burnette, D. T. , & Coffey, R. J. (2019). Reassessment of exosome composition. Cell, 177(2), 428–445.e18. 30951670 10.1016/j.cell.2019.02.029 PMC6664447

[phy215866-bib-0020] Knapp, M. , Tu, X. , & Wu, R. (2019). Vascular endothelial dysfunction, a major mediator in diabetic cardiomyopathy. Acta Pharmacologica Sinica, 40(1), 1–8. 29867137 10.1038/s41401-018-0042-6 PMC6318313

[phy215866-bib-0021] Kordelas, L. , Rebmann, V. , Ludwig, A. K. , Radtke, S. , Ruesing, J. , Doeppner, T. R. , Epple, M. , Horn, P. A. , Beelen, D. W. , & Giebel, B. (2014). MSC‐derived exosomes: A novel tool to treat therapy‐refractory graft‐versus‐host disease. Leukemia, 28(4), 970–973. 24445866 10.1038/leu.2014.41

[phy215866-bib-0022] Laight, D. W. , Carrier, M. J. , & Änggård, E. E. (2000). Antioxidants, diabetes and endothelial dysfunction. Cardiovascular Research, 47(3), 457–464. 10963719 10.1016/s0008-6363(00)00054-7

[phy215866-bib-0023] Leon, B. M. , & Maddox, T. M. (2015). Diabetes and cardiovascular disease: Epidemiology, biological mechanisms, treatment recommendations and future research. World Journal of Diabetes, 6(13), 1246–1258. 26468341 10.4239/wjd.v6.i13.1246 PMC4600176

[phy215866-bib-0024] Luo, B. , Soesanto, Y. , & McClain, D. A. (2008). Protein modification by O‐linked GlcNAc reduces angiogenesis by inhibiting Akt activity in endothelial cells. Arteriosclerosis, Thrombosis, and Vascular Biology, 28(4), 651–657. 18174452 10.1161/ATVBAHA.107.159533 PMC2734484

[phy215866-bib-0025] Ma, J. , Zhao, Y. , Sun, L. , Sun, X. , Zhao, X. , Sun, X. , Qian, H. , Xu, W. , & Zhu, W. (2017). Exosomes derived from Akt‐modified human umbilical cord mesenchymal stem cells improve cardiac regeneration and promote angiogenesis via activating platelet‐derived growth factor D. Stem Cells Translational Medicine, 6(1), 51–59. 28170176 10.5966/sctm.2016-0038 PMC5442756

[phy215866-bib-0026] McLeod, C. J. (2022). Safety evaluation of intracoronary infusion of extracellular vesicles in patients with AMI (EV‐AMI). Available from: https://clinicaltrials.gov/ct2/show/NCT04327635

[phy215866-bib-0027] Nazarie Ignat, S. R. , Gharbia, S. , Hermenean, A. , Dinescu, S. , & Costache, M. (2021). Regenerative potential of mesenchymal stem Cells' (MSCs) secretome for liver fibrosis therapies. International Journal of Molecular Sciences, 22(24), 13292. 34948088 10.3390/ijms222413292 PMC8705326

[phy215866-bib-0028] Ophelders, D. R. M. G. , Wolfs, T. G. A. M. , Jellema, R. K. , Zwanenburg, A. , Andriessen, P. , Delhaas, T. , Ludwig, A. K. , Radtke, S. , Peters, V. , Janssen, L. , Giebel, B. , & Kramer, B. W. (2016). Mesenchymal stromal cell‐derived extracellular vesicles protect the fetal brain after hypoxia‐ischemia. Stem Cells Translational Medicine, 5(6), 754–763. 27160705 10.5966/sctm.2015-0197 PMC4878333

[phy215866-bib-0029] Potz, B. A. , Scrimgeour, L. A. , Pavlov, V. I. , Sodha, N. R. , Abid, M. R. , & Sellke, F. W. (2018). Extracellular vesicle injection improves myocardial function and increases angiogenesis in a swine model of chronic ischemia. Journal of the American Heart Association, 7(12), e008344 Available from: https://www.ncbi.nlm.nih.gov/labs/pmc/articles/PMC6220556/. 29895586 10.1161/JAHA.117.008344 PMC6220556

[phy215866-bib-0030] Qi, J. , Liu, Q. , Reisdorf, R. L. , Boroumand, S. , Behfar, A. , Moran, S. L. , Amadio, P. C. , Gingery, A. , & Zhao, C. (2020). Characterization of a purified exosome product and its effects on canine flexor tenocyte biology. Journal of Orthopaedic Research, 38(8), 1845–1855. 31930553 10.1002/jor.24587

[phy215866-bib-0031] Raposo, G. , & Stoorvogel, W. (2013). Extracellular vesicles: Exosomes, microvesicles, and friends. The Journal of Cell Biology, 200(4), 373–383. 23420871 10.1083/jcb.201211138 PMC3575529

[phy215866-bib-0032] Roefs, M. T. , Sluijter, J. P. G. , & Vader, P. (2020). Extracellular vesicle‐associated proteins in tissue repair. Trends in Cell Biology, 30(12), 990–1013. 33069512 10.1016/j.tcb.2020.09.009

[phy215866-bib-0033] Scrimgeour, L. A. , Potz, B. A. , Aboul Gheit, A. , Liu, Y. , Shi, G. , Pfeiffer, M. , Colantuono, B. J. , Sodha, N. R. , Abid, M. R. , & Sellke, F. W. (2020). Intravenous injection of extracellular vesicles to treat chronic myocardial ischemia. PLoS One, 15(9), e0238879. 32915887 10.1371/journal.pone.0238879 PMC7485873

[phy215866-bib-0034] Scrimgeour, L. A. , Potz, B. A. , Gheit, A. A. , Shi, G. , Stanley, M. , Zhang, Z. , et al. (2019). Extracellular vesicles promote arteriogenesis in chronically ischemic myocardium in the setting of metabolic syndrome. Journal of the American Heart Association, 8(15), e012617 Available from: https://www.ncbi.nlm.nih.gov/labs/pmc/articles/PMC6761642/ 31354010 10.1161/JAHA.119.012617 PMC6761642

[phy215866-bib-0035] Soro‐Paavonen, A. , Zhang, W. Z. , Venardos, K. , Coughlan, M. T. , Harris, E. , Tong, D. C. K. , Brasacchio, D. , Paavonen, K. , Chin‐Dusting, J. , Cooper, M. E. , Kaye, D. , Thomas, M. C. , & Forbes, J. M. (2010). Advanced glycation end‐products induce vascular dysfunction via resistance to nitric oxide and suppression of endothelial nitric oxide synthase. Journal of Hypertension, 28(4), 780–788. 20186099 10.1097/HJH.0b013e328335043e

[phy215866-bib-0036] Tan, Y. , Ichikawa, T. , Li, J. , Si, Q. , Yang, H. , Chen, X. , Goldblatt, C. S. , Meyer, C. J. , Li, X. , Cai, L. , & Cui, T. (2011). Diabetic downregulation of Nrf2 activity via ERK contributes to oxidative stress‐induced insulin resistance in cardiac cells in vitro and in vivo. Diabetes, 60(2), 625–633. 21270272 10.2337/db10-1164 PMC3028364

[phy215866-bib-0037] Tune, J. D. , Goodwill, A. G. , Sassoon, D. J. , & Mather, K. J. (2017). Cardiovascular consequences of metabolic syndrome. Translational Research: the Journal of Laboratory and Clinical Medicine, 183, 57–70. 28130064 10.1016/j.trsl.2017.01.001 PMC5393930

[phy215866-bib-0038] Tüzün, S. , Girgin, F. K. , Sözmen, E. Y. , Menteş, G. , & Ersöz, B. (1999). Antioxidant status in experimental type 2 diabetes mellitus: Effects of glibenclamide and glipizide on various rat tissues. Experimental and Toxicologic Pathology, 51(4–5), 436–441. 10445412 10.1016/S0940-2993(99)80036-0

[phy215866-bib-0039] Um, S. H. , D'Alessio, D. , & Thomas, G. (2006). Nutrient overload, insulin resistance, and ribosomal protein S6 kinase 1, S6K1. Cell Metabolism, 3(6), 393–402. 16753575 10.1016/j.cmet.2006.05.003

[phy215866-bib-0040] Vitale, C. , Mercuro, G. , Cornoldi, A. , Fini, M. , Volterrani, M. , & Rosano, G. M. C. (2005). Metformin improves endothelial function in patients with metabolic syndrome. Journal of Internal Medicine, 258(3), 250–256. 16115299 10.1111/j.1365-2796.2005.01531.x

[phy215866-bib-0041] Vrijsen, K. R. , Maring, J. A. , Chamuleau, S. A. J. , Verhage, V. , Mol, E. A. , Deddens, J. C. , Metz, C. H. G. , Lodder, K. , van Eeuwijk, E. C. M. , van Dommelen, S. M. , Doevendans, P. A. , Smits, A. M. , Goumans, M. J. , & Sluijter, J. P. G. (2016). Exosomes from cardiomyocyte progenitor cells and mesenchymal stem cells stimulate angiogenesis via EMMPRIN. Advanced Healthcare Materials, 5(19), 2555–2565. 27570124 10.1002/adhm.201600308

[phy215866-bib-0042] Wautier, J. L. , Zoukourian, C. , Chappey, O. , Wautier, M. P. , Guillausseau, P. J. , Cao, R. , Hori, O. , Stern, D. , & Schmidt, A. M. (1996). Receptor‐mediated endothelial cell dysfunction in diabetic vasculopathy. Soluble receptor for advanced glycation end products blocks hyperpermeability in diabetic rats. The Journal of Clinical Investigation, 97(1), 238–243. 8550841 10.1172/JCI118397 PMC507085

[phy215866-bib-0043] Xu, C. , Karbasi, C. , Brinck Teixeira, R. , Broadwin, M. , Sellke, F. , & Abid, R. (2023). Diabetic state of human coronary artery endothelial cells results in altered effects of bone mesenchymal stem cell‐derived extracellular vesicles. Available from: https://data.mendeley.com/datasets/sc64ncrgp6 10.14814/phy2.1586638114067

[phy215866-bib-0044] Xu, Z. , Sun, J. , Tong, Q. , Lin, Q. , Qian, L. , Park, Y. , & Zheng, Y. (2016). The role of ERK1/2 in the development of diabetic cardiomyopathy. International Journal of Molecular Sciences, 17(12), 2001. 27941647 10.3390/ijms17122001 PMC5187801

[phy215866-bib-0045] Zhang, B. B. , Zhou, G. , & Li, C. (2009). AMPK: An emerging drug target for diabetes and the metabolic syndrome. Cell Metabolism, 9(5), 407–416. 19416711 10.1016/j.cmet.2009.03.012

[phy215866-bib-0046] Zhang, Y. , Zhang, X. , Zhang, H. , Song, P. , Pan, W. , Xu, P. , Wang, G. , Hu, P. , Wang, Z. , Huang, K. , Zhang, X. , Wang, H. , & Zhang, J. (2021). Mesenchymal stem cells derived extracellular vesicles alleviate traumatic hemorrhagic shock induced hepatic injury via IL‐10/PTPN22‐mediated M2 Kupffer cell polarization. Frontiers in Immunology, 12, 811164. 35095903 10.3389/fimmu.2021.811164 PMC8790700

[phy215866-bib-0047] Zhang, Z. , Apse, K. , Pang, J. , & Stanton, R. C. (2000). High glucose inhibits glucose‐6‐phosphate dehydrogenase via cAMP in aortic endothelial cells. The Journal of Biological Chemistry, 275(51), 40042–40047. 11007790 10.1074/jbc.M007505200

