# Supplementary material for: Diabetic state of human coronary artery endothelial cells results in altered effects of bone mesenchymal stem cell‐derived extracellular vesicles
Source: Physiol Rep. 2023 Dec 19;11(24):e15866. doi: 10.14814/phy2.15866 (PMC10730301; doi:10.14814/phy2.15866)
Supplement: Supplementary file 1 — Data S1. [file PHY2-11-e15866-s001.pdf]

Ruhul Abid, MD, PhD
Associate Professor of Surgery
Brown University Warren Alpert Medical School
Co-Director, Cardiovascular Research Fellowship Program
Cardiothoracic Surgery Division, Rhode Island Hospital
1 Hoppin St, Coro West 5.231, Providence, RI 02903
Email: Ruhul_Abid@brown.edu ; Tel: 401-444-6527

July 7, 2023

Dr. Josephine Adams
Editor-in-Chief
Physiological Reports

Dear Dr. Adams,

I am writing to thank you for the opportunity to consider our research article entitled "Diabetic State of Human Coronary Artery Endothelial Cells Results in Altered Effects of Human Bone Marrow Mesenchymal Stem Cell-derived Extracellular Vesicles," by Xu et al. for consideration to be published as an original research paper in your esteemed journal, Physiological Reports.

Progenitor cell-derived extracellular vesicles (EV) have been used successfully in animal models of ischemic cardiovascular disease for many years now, but has had dampened effects in models of metabolic syndrome. To transition the use of EVs successfully into clinical practice, this barrier needs to be addressed. Here, we studied the effects of EVs on non-diabetic and diabetic human coronary artery endothelial cells (HCAEC), and found that while the EVs had their expected regenerative and anti-inflammatory effects in the non-diabetic cells, the full effects were not attained in the diabetic cells. We identified several metabolic abnormalities in the diabetic endothelial cells that could explain these differences, including a heightened and metabolic inflammatory state and differential stress/starvation and proliferative signaling.

To the best of our knowledge, attempts in using EVs effectively in a clinical setting are ongoing, but achieving a meaningful clinical benefit may be difficult as human patients often have many co-morbidities in conjunction with metabolic syndrome. Based on our findings in this study, for EVs to achieve their full clinical benefits, we believe that treatment of metabolic syndrome is likely necessary. We hope that you will share our enthusiasm as the findings reported here will improve efforts on making EVs a clinically viable option for patients with ischemic cardiovascular disease.

Lastly, we confirm that neither the manuscript nor any parts of its content are currently under consideration or published in another journal, and all authors have approved the manuscript and agree with its submission to Physiological Reports.

We thank you for your consideration.
Sincerely yours,

Ruhul Abid, MD, PhD
